# Targeting the dermis for melasma maintenance treatment

**DOI:** 10.1038/s41598-023-51133-w

**Published:** 2024-01-10

**Authors:** Hee Jeong Han, Jin Cheol Kim, Young Joon Park, Hee Young Kang

**Affiliations:** Department of Dermatology, Ajou University Hospital, 164, World Cup-Ro, Yeongtong-Gu, Suwon-Si, Gyeonggi-Do 16499 South Korea

**Keywords:** Health care, Medical research

## Abstract

Melasma relapse is almost common after discontinuation of conventional treatment. Recent studies suggesting that photoaging dermis is the main pathomechanism of melasma, emphasize the dermal targeting therapy. We investigated maintenance effect of microneedling radiofrequency (RF) for melasma treatment. Subjects with melasma were administered oral tranexamic acid and triple combination cream for 2 months and a randomly assigned half face was treated with RF. After discontinuation of conventional therapy, the half face RF continued monthly over 6 months. Modified melasma area severity index (mMASI) score and L* value by a chromameter were collected monthly. Fifteen subjects were enrolled and eleven completed the 8-month study. At 2nd month of conventional therapy, all subjects showed improvement with a 64% reduction in mMASI score. With continuous RF treatment, the improvement was well maintained; whereas in untreated side, the Δ L* gradually decreased, returning to the baseline after the conventional therapy ended. The continuous microneedling RF therapy is beneficial in maintaining the conventional therapy of melasma suggesting the protective effect of dermal targeting therapy in melasma development.

(Clinical Trial registration number: NCT05710068, date of first registration: 02/02/2023).

## Introduction

Melasma is a common acquired hyperpigmentary disorder and the treatment remains challenging^1^. The gold standard treatment for melasma is Kligman’s formulation, which comprises hydroquinone, retinoid and corticosteroid^2^. Recent studies have shown that oral administration of tranexamic acid (TA) has emerged as a promising treatment for melasma^3–5^. Furthermore, the combination treatment was more effective than the topical agent alone in a randomized controlled trial^6^. However, relapse after discontinuation of these combination treatments is almost common and remains a major challenge in melasma treatment.

Melasma is recently regarded as one of the phenotypes of photoaging^7^. Evolving research indicates more heterogeneous pathomechanisms observed in melasma, including solar elastosis, increased vascularization, and an increased number of senescent fibroblasts, in addition to the inappropriate melanocyte activation^7,8^. The continuous melanogenic signaling from the aging dermis, including photoaged fibroblasts or increased vasculatures, activates melanocytes and contributes to increased pigmentation in melasma^9^. Gene analysis studies, on aging pigmented skin, have identified the following novel melanogenesis-modulating factors from UV-radiated fibroblasts: secreted frizzled-related protein-2 (sFRP2)^10^, stromal-derived factor 1 (SDF1)^11^, and growth differentiation factor-15 (GDF15)^12^. These factors result in increased skin pigmentation and influence the development of pigmentary diseases associated with photoaging. Taken together, all these observations suggest that a dermal targeting strategy should be considered for melasma treatment apart from the direct controlling tyrosinase of melanocytes. In this prospective, split-face study, we investigated the maintenance effect of dermal targeting therapy using radiofrequency (RF) microneedling after conventional melasma treatment with oral TA and triple combination cream (TCC).

## Results

### Subject demographics and flow diagram

**Figure 1:**
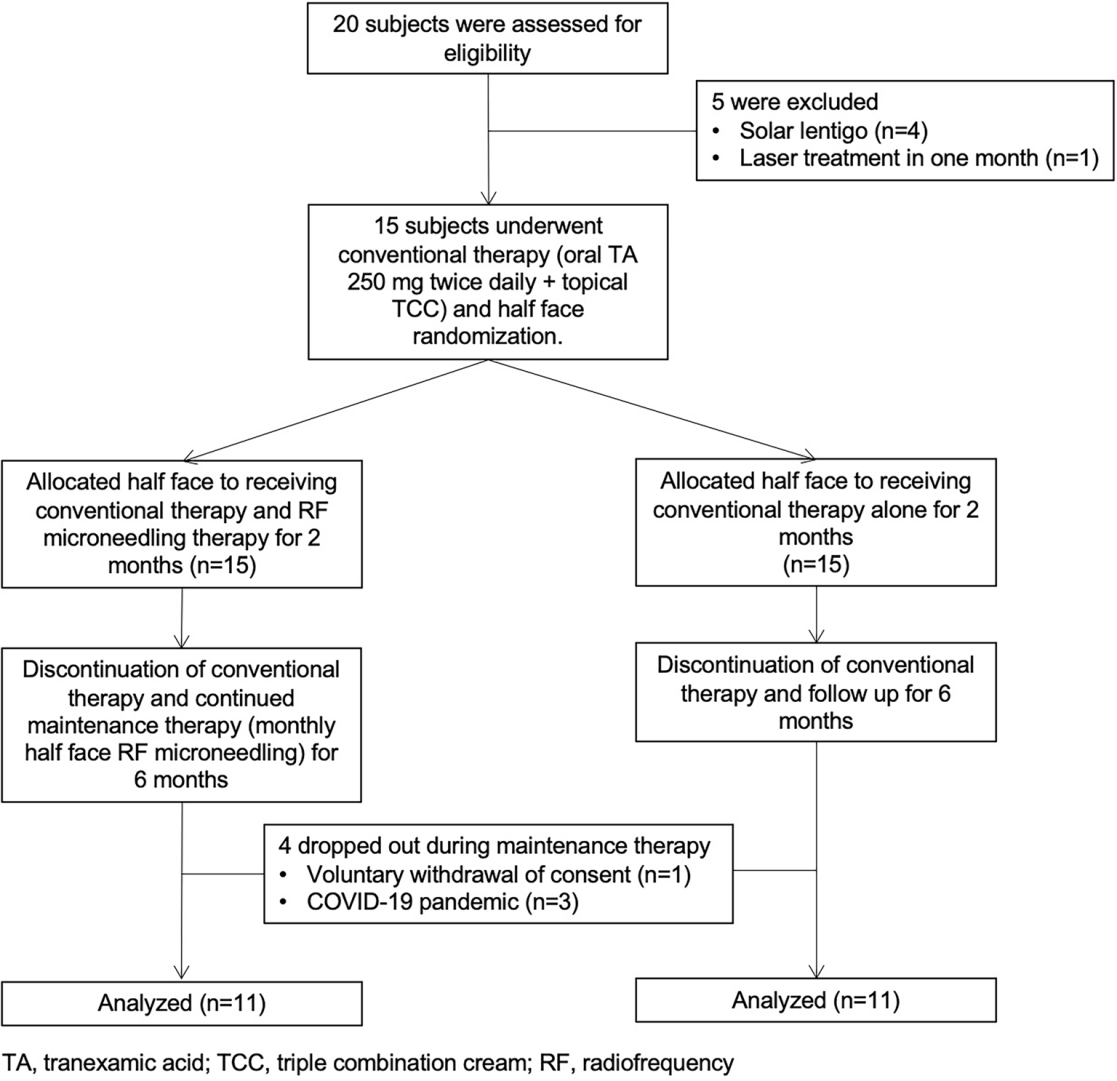
Flow diagram of the study.

This study has been registered in a Clinical Trial. (Clinical Trial registration number: NCT05710068, date of first registration: 02/02/2023) A total of fifteen female volunteers with facial melasma were analyzed. All the subjects had the Fitzpatrick skin type III (n = 5) or IV (n = 10). The mean age was 47.07 years (range 37–62 years) and the average duration of melasma was 9.73 years. The baseline mMASI score was 5.02 ± 2.27 and L* values of the RF treated side and untreated side were 59.79 and 60.44, respectively (Table 1). All subjects received the conventional therapy of oral TA and TCC. The half face was randomly assigned for RF treatment (right side, n = 9; left side, n = 6). All subjects completed 2-month therapy and participated in the maintenance therapy with RF; however, four subjects withdrew during the maintenance therapy because of voluntary withdrawal of consent (n = 1) and COVID-19 pandemic (n = 3). Eventually, eleven subjects completed the study. The flow diagram of the study is shown in Fig. 1.

**Table 1:** Baseline demographics of subjects.

	Conventional therapy^1^ (N = 15)		Maintenance therapy^2^ (N = 11)	
	Mean	SD	Mean	SD
Age (years)	47.07	7.83	47.27	8.46
Duration of melasma (years)	9.73	5.22	10.36	5.92
Baseline mMASI^1^ score	5.02	2.27	5.27	2.22
Baseline L* value				
RF treated side	59.79	1.79	60.54	1.18
RF untreated side	60.44	1.91	60.94	1.76
After 2-month L* value				
RF treated side	62.86	1.74		
RF untreated side	62.55	2.03		

*SD* standard deviation, *mMASI* modified Melasma Area and Severity index, *RF* Radiofrequency microneedling.

^1^Conventional therapy: oral tranexamic acid 250 mg twice daily with topical triple combination cream once a day for 2 months.

^2^Maintenance therapy: radiofrequency microneedling treatment on the randomly assigned half face monthly for 6 months.

### Oral tranexamic acid combined with triple combination cream improves melasma

**Figure 2:**
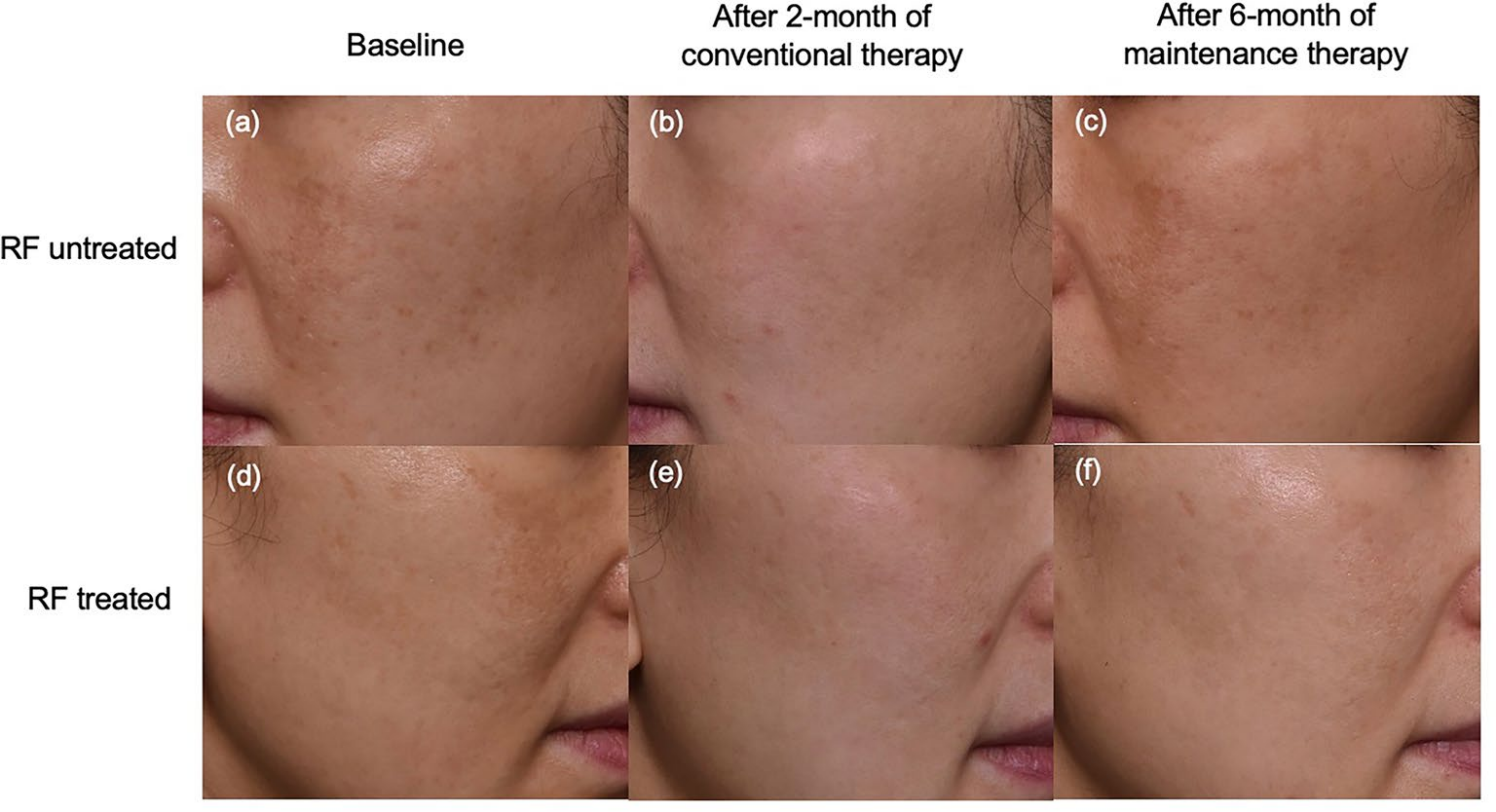
Efficacy of conventional and maintenance treatments in a 46-year-old woman with melasma. The combination therapy of oral tranexamic acid and triple combination cream markedly improved the melasma (**a**–**e**). The improvement maintained on the radiofrequency treated side (**f**) after discontinuation of conventional therapy, whereas it returned to the baseline on the untreated side (**c**).

All the 15 subjects showed marked improvement after 2-month conventional treatment. (Fig. 2a–e) The mMASI score significantly decreased from 5.02 ± 2.27 to 1.80 ± 1.38 (p < 0.01) with 64.1% reduction on average. Both L* values of the RF treated and untreated side increased from 59.79 ± 1.79 and 60.44 ± 1.91 to 62.86 ± 1.74 and 62.55 ± 2.03, respectively (3.07 vs. 2.11, p = 0.40). From the perspective of the synergistic effect of RF, there was no significant difference in the RF group in addition to the combination treatment of oral TA and TCC, although RF group showed better improvement. There were no significant side effects including erythema or irritation.

### The continuous adjuvant RF treatment helps maintaining the treatment effect of conventional therapy

**Figure 3:**
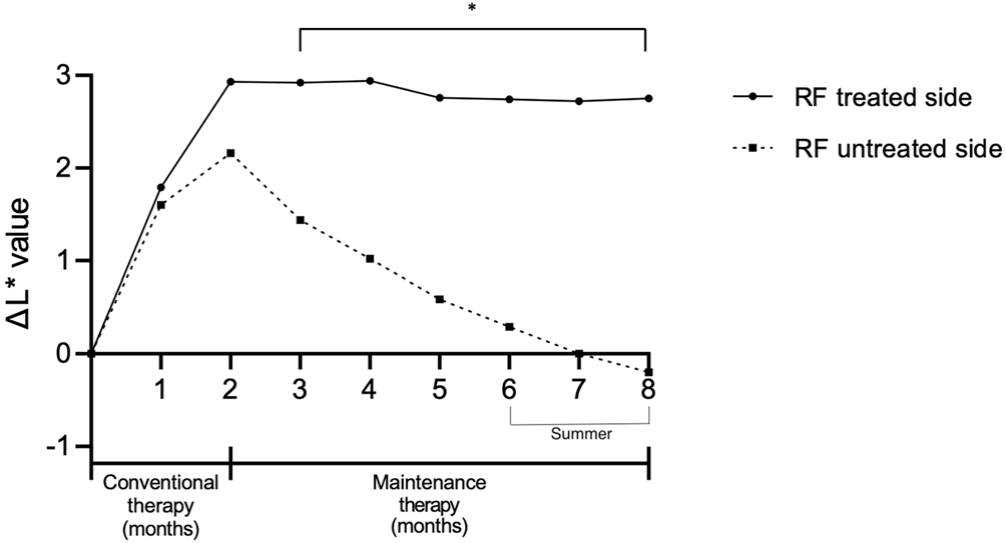
The continuous radiofrequency treatment maintains the treatment effect of conventional therapy. After discontinuation of conventional therapy, Δ L* on RF treated side maintained above 2.7 through the study duration *p < 0.01.

After discontinuation of conventional therapy, a total of eleven subjects maintained the half face RF every month for 6 months. The Δ L* (mean change of L* value from the baseline) of the untreated side decreased significantly from the first month after cessation of conventional therapy. The mean L* value reached to the baseline in 6 months (Figs. 2c,f, 3). In contrast, the RF treated side helped maintaining the treatment effect after cessation of the 2-month previous treatment, i.e. Δ L* on the RF treated side maintained above 2.7 through the study duration. Mild erythema and pain were reported on the area that were treated with RF therapy but resolved in 30 min. Other serious adverse effects such as bleeding, infection, postinflammatory hyperpigmentation and scarring were not reported during the study period.

## Discussion

The present study showed that dermal targeting therapy using RF microneedling can prevent the relapse of previously improved melasma lesions. In the untreated side, seven subjects (63.6%) returned to the baseline state at 6 months follow-up after discontinuation of the conventional treatment, whereas RF treated side maintained the improvement of melasma, suggesting the protective effect of RF in melasma relapse. It should be noted that in this study, the subjects were enrolled at the end of winter season and followed up after summer to remove confounding factor of spontaneous improvement of melasma during autumn and winter. Indeed, the adjuvant RF treatment helped maintaining the efficacy, even though they went through 1 summer. However, the microneedling RF in addition to the conventional therapy did not show superiority to the conventional therapy alone, although former showed better improvement in the Δ L* value. The reason is likely because the effects of oral TA and TCC are already quite substantial. Indeed, a previous report has shown that the microneedling RF has a synergistic effect on the aging-associated pigmentary diseases in combination with the conventional Q-switched ND:YAG laser^13^.

The dermal targeting therapy involves energy-based devices such as RF, pulsed-dye laser and intense pulsed light to induce dermal rejuvenation^14^. In this study, the microneedle RF device was chosen to manipulate only dermal structures, in which the microneedles generate thermal coagulation columns in the dermis, not in the epidermis^15^. The RF treatment induced rejuvenation of aging dermis in melasma and senile lentigo, which subsequently makes it possible to resolve inappropriate melanin production from the dermis^11,16^. A nonspecific reduction in p16^INK4A^ positive senescent fibroblasts and the stimulation of procollagen production after RF treatment were shown to contribute to a decrease in epidermal pigmentation in melasma and senile lentigo^11,16^. The beneficial effect of pulsed dye laser for melasma was also previously shown^17^. The reduction of dermal vasculatures of melasma appeared to improve the treatment effect of topical TCC and also prevented the relapse of melasma^17,18^. This effect was thought to be related in reduction of melanocyte activation signaling from endothelial cells such as endothelin-1 and stem cell factor^19,20^. Taken together, it is speculated that the dermal targeting treatment might be beneficial for long-term treatment of melasma. However, particular attention should be given to the possibility of worsening melasma due to epidermal heating, especially in non-insulated RF therapy.

Another interesting finding of our study include that the effectiveness of oral TA combined with TCC for melasma treatment was confirmed. All the subjects showed marked improvement of mMASI (64.1% reduction) after 2-month treatment that is comparable with previous studies. There was 65.45% improvement in mMASI at week 8^21^ and 62% improvement at week 20^22^ in Mexican study, 51% improvement in MASI at week 12 in Iran study and 87.9% improvement in MASI at week 8 in Indian study^23^ after administering oral TA and TCC. The efficacy of combination treatment is superior to the monotherapy of oral TA, i.e. oral TA alone showed 49% reduction in mMASI score for 3-month treatment^5^. Moreover, no serious adverse events were reported in the conventional treatment^5,21,23,24^. Taken together, the combination therapy of oral TA and TCC might be considered as a standard treatment of melasma. However, relapse after cessation of the treatments is remained as a major challenge. In the presented study, we also found that the efficacy gradually disappears after discontinuation in 1 month and all returned to the baseline in 6 months. This recurrence rate is similar to the previous studies; most patients who received oral TA 500 mg daily experienced relapse in 6 weeks post discontinuation^25^. Relapse rate after ceasing oral TA was reported 72% within 2 months^26^ and mean MASI was rebound to 77.4% compared to baseline within 3 months after discontinuation of oral TA and topical hydroquinone^27^. Therefore, these finding emphasize the beneficial role of dermal targeting therapy for the melasma treatment.

Our study has some limitations; there was a small sample size, and all the subjects were female with similar ethnic backgrounds. However, the strength of this study is the first study investigating the long-term maintenance effects of dermal targeting therapy using microneedling RF for melasma treatment.

In conclusion, the microneedling RF therapy appears to be beneficial in combination with the conventional therapy of melasma, suggesting the protective effect of dermal targeting therapy in melasma relapse.

## Materials and methods

### Study design

This was a randomized, split-face clinical trial conducted at the department of dermatology in a single tertiary center (Ajou University Hospital, Seoul, Korea) from December, 2021 to August, 2022. This study was divided into two stages; first 2 months, oral TA and TCC as well as the half face RF treatment were given. After the improvement of melasma, oral TA and TCC were discontinued, but the RF microneedling continued on half of the face for an additional 6 months.

### Subjects

Melasma was clinically diagnosed by three dermatologists (J.C. Kim, Y.J. Park, H.Y. Kang). Subjects who had undergone prior aesthetic medical procedures or used topical depigmenting agents in three months prior to the study and were pregnant or lactating were all excluded. A total of 20 volunteers were screened and five subjects failed screening; four were diagnosed as solar lentigo and one had a history of laser treatment in one month. As a result, fifteen female volunteers with facial melasma were enrolled in the study. Four subjects withdrew during the study because of 1 voluntary withdrawal of consent and 3 COVID-19 pandemics. Eleven subjects finally completed the 8-month study.

### Intervention

**Figure 4:**
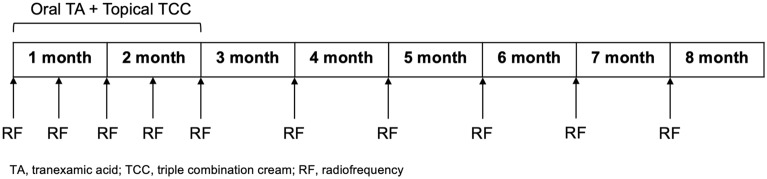
Timeline of the intervention in the study.

For the first 2 months, the subjects were instructed to take oral TA 250 mg twice daily and apply TCC composed of 5% hydroquinone, 0.003% tretinoin, and 1% hydrocortisone (Melanon cream, DONG-A ST, Korea) to the entire face once a day for 8 weeks. This regimen was referred to as “conventional therapy” in this study. Concurrently, randomly assigned half face was treated using a pulsed-type RF device in bipolar mode with 25 non-insulated microneedles (Sylfirm X™, VIOL, Korea) every 2 weeks. Parameters were set pulsed-wave mode (PW2) at level 4–6 targeting mild erythema with a penetration depth of 0.3 mm for two passes and 0.8 mm for one pass. After discontinuation of the conventional therapy, the half face RF therapy was maintained every month for 6 months. This regimen was referred to as “maintenance therapy”. (Fig. 4.)

All subjects received sunscreens (Uvidea Anthelios, LA ROCHE-POSAY, Paris) and barrier repair creams (Zeroid intensive cream, NEOPHARM, Korea) during the study. All the treatments were performed by a single experienced dermatologist. Standard clinical photographs were taken every month with a digital camera (Nikon D5600, Japan) and a pigment imaging tool (JANUS Pro Hybrid®, PIE Co., Ltd., Korea).

### Treatment outcomes

Modified melasma area severity index (mMASI) score^28^ and L* value measured by a chromameter were collected monthly. The mMASI score was assessed by two independent dermatologists (H.J. Han and H.Y. Kang) using digital photographs every month as follows: forehead, 0.15 × D × A, malar, 0.30 × D × A, chin, 0.05 × D × A; A, area involved; D, darkness. Skin pigmentation levels were measured on both sides of the face respectively using a chromameter (CR-300; Minolta, Japan), and values of lightness denoted by L* were recorded every month. The measured location was recorded to compare the lightness of the same spot. The Δ L* (mean change of L* value from the baseline) of the RF treated side and untreated side were calculated respectively every month. Adverse effects were collected at each treatment session and follow-up visit.

### Statistical analysis

Data were evaluated by two-way repeated measures analysis of variance (ANOVA) to compare the trends of L* values. The result was considered statistically significant when the p value was less than 0.05 and SPSS software (IBM SPSS Statistics for Windows, Version 25.0. Armonk, NY) was used to analyze the data.

### Ethical statement

This study was approved by the institutional review board of Ajou University Hospital (IRB number: AJIRB-DEV-INT-21-133). The protocol was initiated following written informed consent in adherence to the principles of Declaration of Helsinki and regulations for Good Clinical Practice. (Clinical Trial registration number: NCT05710068, date of first registration: 02/02/2023).

## Data Availability

The datasets generated and analysed during the current study are not publicly available due to clinical photos containing personal information but are available from the corresponding author on reasonable request.

## Abbreviations

GDF15: Growth differentiation factor-15
mMASI: Modified melasma area severity index
RF: Radiofrequency
SDF1: Stromal-derived factor 1
sFRP2: Secreted frizzled-related protein-2
TA: Tranexamic acid
TCC: Triple combination cream
